# Discovering the 60 years old secret: identification of the World War II mass grave victims from the island of Daksa near Dubrovnik, Croatia

**DOI:** 10.3325/cmj.2011.52.327

**Published:** 2011-06

**Authors:** Igor Borić, Jelena Ljubković, Davorka Sutlović

**Affiliations:** 1 General Hospital Dubrovnik, Department of Pathology, Dubrovnik, Croatia; 2 University Hospital Split and School of Medicine Split, Department of Forensic Medicine, Split, Croatia

## Abstract

**Aim:**

To describe the organization, field work, forensic anthropological examination, and DNA analysis conducted to identify the victims from a World War II mass grave found on the Dalmatian island of Daksa near Dubrovnik (Croatia) in 2009.

**Methods:**

Excavation of the site was performed according to standard archeological procedures. Basic anthropological examination was made to determine the minimum number of victims, sex, age at death, and height. The bones with pathological and traumatic changes were identified. DNA was extracted from powdered bones and relatives’ blood samples. Y-chromosome and autosomal short tandem repeats (STR) were used to establish the relationship of the remains with the putative family members.

**Results:**

The remains were found to belong to at least 53 distinctive victims. All were male, mostly with gunshot wounds to the head. DNA analysis and cross-matching of the samples with relatives resulted in 14 positive identifications using the Y-chromosomal STRs and 4 positive identifications using the autosomal STRs.

**Conclusions:**

This study showed that even in cases of more than 50-year-old, highly degraded human remains from mass graves, Y-chromosomal and autosomal STRs analysis can contribute to identification of the victims.

After the partisan (communist-led) forces had entered Dubrovnik on October 18, 1944, mass executions followed, among them the execution on the nearby island of Daksa (Figure 1 and 2). According to historical sources (1), the execution took place on October 25, 1944 and the following days. According to the verdict issued by the “Court of the Military Command for the South Dalmatian Region” and announced on a poster on October 29, 1944, 36 Dubrovnik citizens, most of them prominent intellectuals, were “sentenced to be shot by a firing squad.” The same historical sources do not state whether a trial was held or if there was a court under such a name (1).

**Figure 1:**
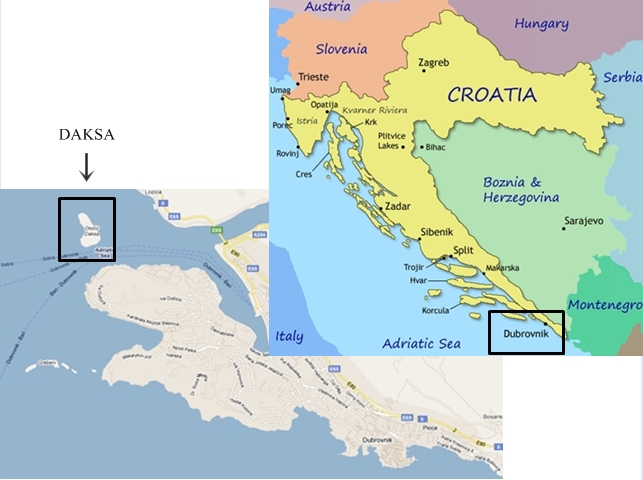
Map of Croatia showing its position in Europe and Dubrovnik with its nearby island of Daksa.

**Figure 2:**
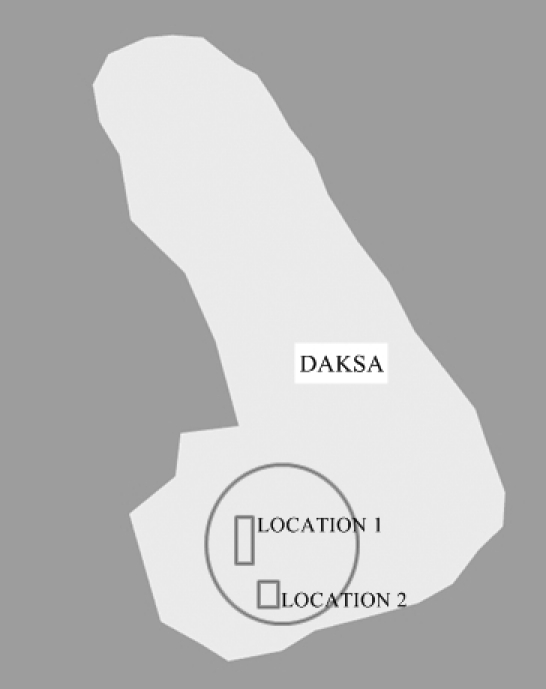
Map of the island of Daksa showing two sites of the mass grave, Location 1 and Location 2.

In forensics, if the recovery of skeletons takes places a long time after death the possibilities to obtain information about victims’ clothing, personal belongings, sex, age, height, and other characteristics may often be reduced. In such cases, DNA typing techniques may provide useful information. Y-short tandem repeat (STR) markers are paternally inherited and allow identification of male missing persons when the only available reference is a male paternal relative. The autosomal STR DNA profiling was established by using the commercially available miniSTR assay, which proved useful to obtain information from ancient and degraded DNA samples and was used in cases when the only available reference was a missing person’s daughter (2). The DNA technology, including both STR analysis and mitochondrial DNA analysis, was already confirmed as a method of choice in the identification of missing persons in the 1991-1995 war in Croatia (3,4).

The aim of this study was describe the organization, field work, forensic anthropological examination, and DNA analysis by chromosome and autosomal STRs conducted to identify the victims from a World War II mass grave on the island of Daksa.

## Materials and methods

**Figure 3:**
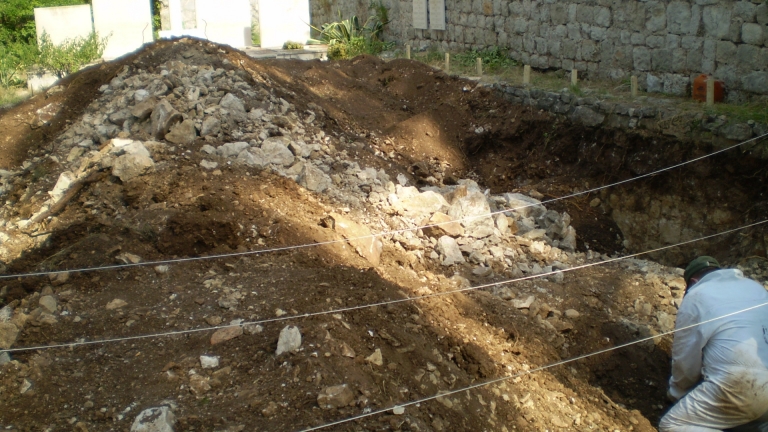
Mass grave site.

**Figure 4:**
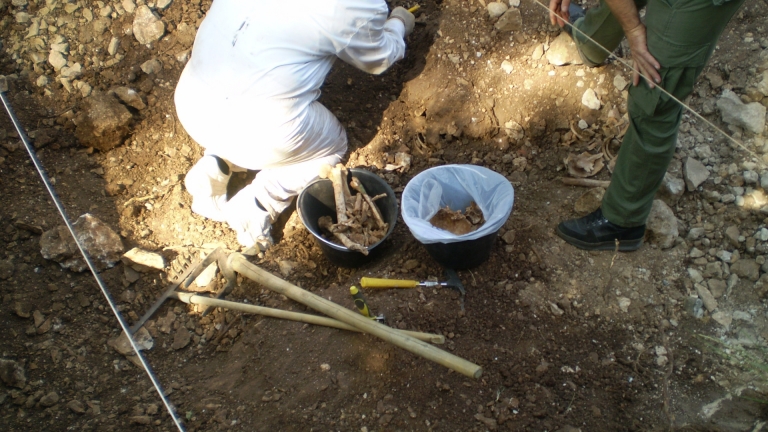
Mass grave exhumation.

The field work took place over fewer than 5 working days. During that time (including the time of departure/return from the island), the site was marked and trial trenches were dug and two locations treated (surface larger than 80 m^2^, more than 150 m^3^ of the excavated material) (Figure 3). Approximately 10 000 bones and skeletal fragments, and a number of items were discovered, packed, and transported (Figure 4). In such circumstances, in most cases it was impossible to determine with certainty the body position and their interrelations.

### Field work

The exhumation of mortal remains on the island of Daksa started on September 24, 2009, pursuant to the Order of the County Court in Dubrovnik. According to the historical data (1), it was assumed that the location of the mass grave was in the ruins of the former farm building basement. Another mass grave was also believed to be in the immediate vicinity. The second location was discovered about 15 m away from the first location.

**Figure 5:**
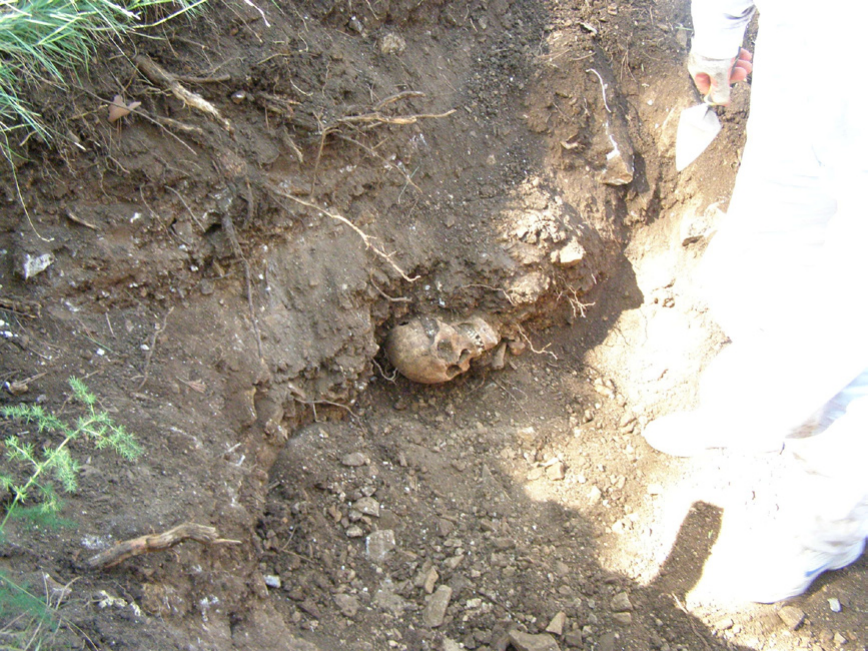
Human skeletons in the mass grave after the surface layer of the soil was removed by a bulldozer.

Location 1 was an area surrounded by remains of a wall. It was divided, labeled, and staked out by sticks and ropes into 55 equal fields measuring approximately 1 × 1 m. The surface layer of the soil was removed by a bulldozer. Bones started to emerge at the depth of approximately 1 m from the surface (Figure 5). Due to a large number of mixed and separated bones in different positions, intermingled with large stones, it was decided to give up the skeleton presentation in situ. This primarily referred to the Location 1, fields labeled “d.” The bones were carefully excavated. After having defined the orientation (cranial/caudal) and the position related to the marked fields and the layer depth, the bones were packed in labeled cardboard boxes.

**Figure 6:**
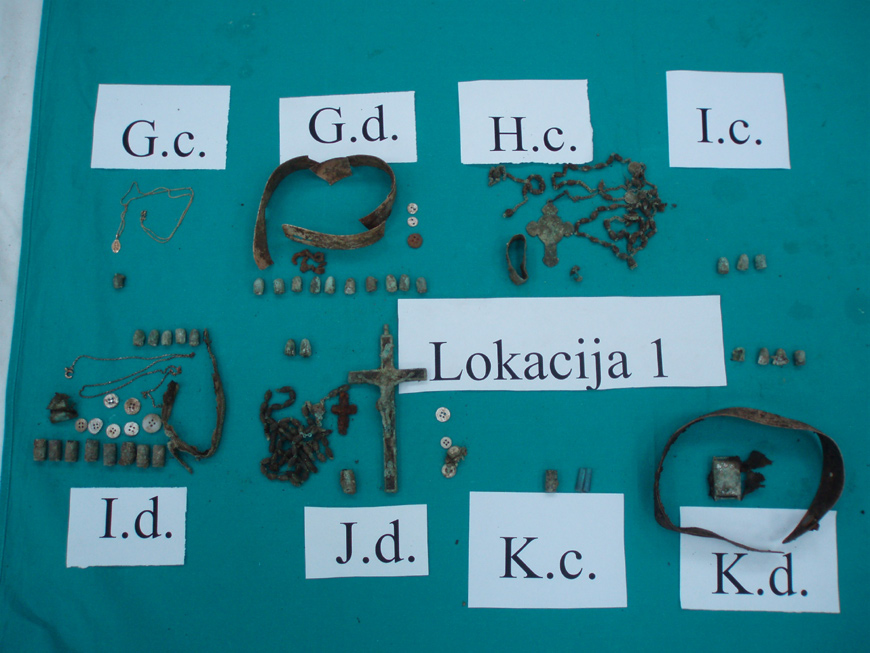
The objects found at the site.

Location 2 was not staked out as Location 1, but was shaped as the excavation went on. Three parallel trenches were dug and marked as 2, 3, and 4, and divided into 9 equal fields measuring approximately 1 × 1 m. The trenches followed the direction of cracks in limestone. The findings of the excavation were the same as those at Location 1. The work was additionally obstructed by thick pine roots. Various items were found along with the bones at both locations: fragments of priestly collars, rosaries, buttons, necklaces, bullets, and shells (Figure 6). The excavation went on up to the hard layer at the depth of about 2 m.

### Work at the pathology room

The remains were transferred to the Department of Pathology of the Dubrovnik General Hospital, where they were cleaned and sorted out. Where possible, the bones were placed in anatomical position and analyzed to determine the age, sex, height, and changes that had occurred during lifetime or after death, and photographed. Samples for the DNA analysis were taken subsequently. After having taken measures of thighbones and head diameters, the height of a person was determined by the Trotter equation for calculating the body height (5).

Boundary values for the sex determination, based on the long bone length, were determined as described by Šlaus et al (6). Where possible, sex was determined by pelvic bones and skull characteristics. Due to the significant level of damage, this was generally not possible. Also, because of a large number of bones in a limited area and in the same layer, it was impossible to determine with certainty the body parts that belonged to the same person.

The age was determined by the pelvic articular surface (symphysis) following the Suchey Brooks method (7). Since most of the bones were badly damaged, a reliable determination was generally not possible. The location and the area of discovery and the total number of the bones discovered are shown in Table 1 and Table 2. Skeletal remains were identified and classified according to macro-sites (Location 1 and 2) and micro-sites (marked fields-quadrants) in which they were found (eg, Location 1 field Da). Fields in which skeletal remains were not found are not listed in the tables.

**Table 1:** Archeological data from the mass grave at Daksa Island, Location 1. The data are divided according to marked fields, see Figure 7

Field	Scapula	Clavicula	Humerus	Ulna	Radius	Ribs	Sternum	Vertebrae	Femur	Pelvis 1/2	Sacrum	Tibia	Fibula	Small bones	Skull
**Da**	2	2	2	2	2	20	1	20	1	1	0	3	4	82	1
**Ea**	3	3	3	4	3	29	2	36	5	5	3	3	3	155	1
**Fa**	3	3	3	2	3	33	1	32	0	1	1	0	0	80	2
**Ga**	0	0	0	0	0	1	0	4	2	0	0	4	4	97	2
**Ha**	5	6	6	6	6	84	3	59	6	6	3	2	2	176	0
**Ia**	0	0	0	0	0	0	0	6	2	0	0	3	4	68	1
**Ja**	2	2	2	1	1	19	1	28	1	4	2	0	0	63	1
**Ka**	2	2	2	2	2	17	1	19	0	0	0	0	0	26	1
**Ib**	0	0	0	0	0	0	0	0	0	0	0	2	1	15	0
**Jb**	0	0	0	1	1	0	0	0	1	0	0	1	0	0	0
**Total**	**17**	**18**	**18**	**18**	**18**	**203**	**9**	**204**	**18**	**17**	**9**	**18**	**18**	**762**	**9**
**Fc**	0	0	0	0	0	7	0	7	4	3	1	5	6	0	0
**Gc**	4	4	3	1	1	31	1	23	1	3	2	1	1	81	1
**Hc**	7	4	6	6	6	52	5	63	10	6	6	3	3	200	1
**Ic**	3	3	5	3	4	33	2	33	1	4	2	0	0	93	2
**Kc**	5	4	5	6	5	75	2	58	0	3	3	0	0	188	2
**Fd**	0	0	0	0	0	0	0	1	4	1	1	3	3	13	0
**Gd**	4	4	6	7	6	81	4	58	8	7	1	14	14	281	3
**Hd**	4	5	3	3	5	59	2	69	3	4	2	4	4	175	3
**Id**	7	7	7	9	8	60	2	71	3	3	1	12	13	428	5
**Jd**	4	4	3	2	3	68	2	51	12	6	4	4	2	86	2
**Kd**	6	7	8	6	8	62	3	65	0	3	0	0	0	173	4
**Total**	**44**	**42**	**46**	**43**	**46**	**528**	**23**	**499**	**46**	**43**	**23**	**46**	**46**	**1718**	**23**
**Overall**	**61**	**60**	**64**	**61**	**64**	**731**	**32**	**703**	**64**	**60**	**32**	**64**	**64**	**2480**	**32**

**Table 2:** Archeological data from the mass grave at Daksa Island, Location 2. The data are divided according to marked fields (No. 2-5), see Figure 8.

Field	Scapula	Clavicula	Humerus	Ulna	Radius	Ribs	Sternum	Vertebrae	Femur	Pelvis 1/2	Sacrum	Tibia	Fibula	Small bones	Skull
**2**	2	3	4	1	3	23	1	31	3	3	2	2	2	25	2
**2a**	2	3	3	4	4	35	2	43	5	5	2	5	4	75	0
**2b**	2	2	1	2	2	27	1	23	0	0	0	4	6	231	2
**2c**	2	0	2	1	1	4	0	13	4	10	5	7	6	28	0
**2d**	3	3	3	3	3	57	2	55	6	1	0	0	0	35	2
**2e**	6	5	3	3	3	37	3	35	0	0	1	0	0	76	2
**2f**	1	3	3	4	4	27	1	34	2	1	0	2	2	89	2
**Total**	**18**	**19**	**19**	**18**	**20**	**210**	**10**	**234**	**20**	**20**	**10**	**20**	**20**	**559**	**10**
**3**	2	3	2	2	2	14	0	21	2	2	1	6	8	181	3
**3a**	4	3	3	4	3	37	2	40	7	2	1	6	4	69	0
**3b**	2	1	3	3	4	36	2	28	5	10	4	1	1	115	1
**3c**	6	7	6	3	3	68	3	66	0	0	1	1	0	57	2
**3d**	0	0	0	2	2	4	0	9	0	0	0	0	1	17	1
**Total**	**14**	**14**	**14**	**14**	**14**	**159**	**7**	**164**	**14**	**14**	**7**	**14**	**14**	**439**	**7**
**4**	6	6	5	3	4	69	3	36	4	5	2	2	1	67	2
**4a**	0	0	0	1	2	0	0	6	2	0	0	4	5	84	1
**Total**	**6**	**6**	**5**	**4**	**6**	**69**	**3**	**42**	**6**	**5**	**2**	**6**	**6**	**151**	**3**
**5**	0	0	1	1	0	0	0	0	0	0	0	1	0	3	1
**Overall**	**38**	**39**	**39**	**37**	**40**	**438**	**20**	**440**	**40**	**39**	**19**	**41**	**40**	**1152**	**21**

**Figure 7:**
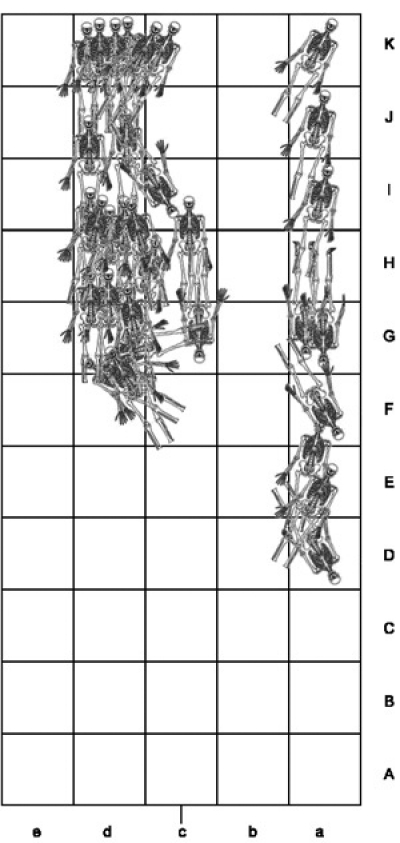
Schematic presentation of the finds at Location 1.

**Figure 8:**
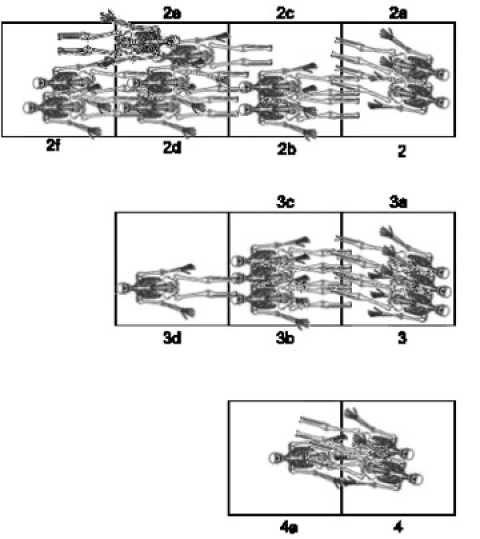
Schematic presentation of the finds at Location 2.

We made a schematic presentation of the assumed position of a particular body in relation to other bodies and their spatial orientation within the labeled schematic site (Figure 7 and 8).

### DNA analysis

Prior to the DNA extraction, the bone/tooth surface was cleaned by abrasion with a grinding tip and sandpaper, the bone/tooth was crushed into small fragments, and stored in sterile polypropylene tubes at -20°C until analysis. After this initial treatment, the DNA extraction was performed as described by Alonso et al (8). At least two independent extractions were done (one or two craniums that presented teeth were sampled, as well as different postcranial bones like femurs). The DNA from blood and bloodstain reference samples of the living relatives was extracted by standard Chelex 100 protocols (9). The polymerase chain reaction (PCR) amplifications were performed on Parkin-Elmer thermal Cycler 9700, using the AmpFiSTR Yfiler PCR Amplification Kit (Applied Biosystems, Foster City, CA, USA) and MiniFiler PCR Amplification Kit (Applied Biosystems) (10,11). Typing of PCR products was performed on the ABI Prism 310 Genetic Analyzer (Applied Biosystems) with the Data Collection Software. Electropherogram data were analyzed with the GeneMapper ID Software, version 3.2 (Applied Biosystems). The internal standard was Liz-500 (Applied Biosystems).

### Analysis of typing results

DNA profiles from skeletal remains were analyzed and compared with DNA profiles of the living relatives. The database was kept in the Microsoft Access 2000 (Microsoft, Seattle, WA, USA). Microsoft Excel 2000 (Microsoft) was used for statistical calculation. Calculation for statistical probability of biological relationship was performed according to standard protocols and the data from the Y Chromosome Haplotype Reference Database (YHRD) database (2,12,13).

## Results

Human remains were found at two sites designated as Location 1 and Location 2, and the skeletal remains belonged to at least 53 persons (Table 1 and 2). It can be stated with certainty that the bones were human and more than 50 years old, the time corresponding to the World War II. The skeletal remains had distinctive male anthropomorphic characteristics. Due to the bone damage of the remains, the age of a certain number of persons could not be determined.

Many of them had metal fillings, gold, and silver teeth and dental bridges and dentures.

**Figure 9:**
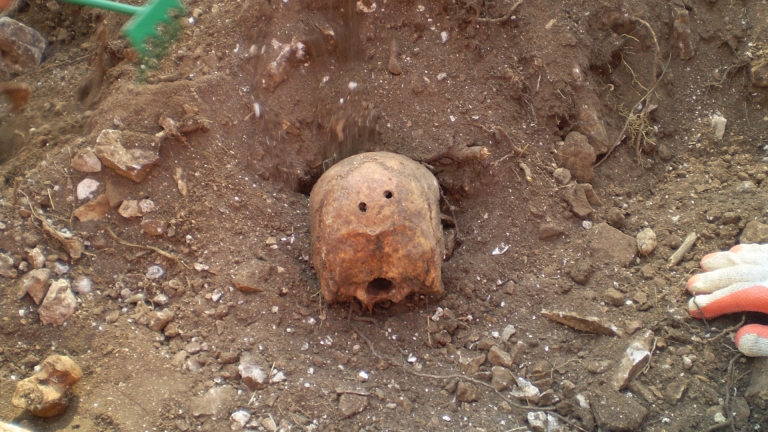
Gunshot wounds in the occipital region of a victim.

Gunshot wounds were identified in 22 persons. Practically all identified entry gunshot wounds were localized in the occipital region (Figure 9). One gunshot wound in the occipital region was identified in 15 persons, 2 gunshot wounds in 5 persons, and 3 gunshot wounds in 2 persons. Only one gunshot wound was identified in an area other than the skull, ie, the hip socket. Many skulls were found in fragments with peri-mortem fractures, although the head gunshot wound was not found. A tibia bone with a peri mortem fracture was also found on the site.

DNA was successfully extracted from 49 out of 53 skeletal remains (Table 3). In each case of tooth and bone samples, several DNA extractions were performed. AmpFiSTR Yfiler PCR Amplification Kit produced 34 out of 49 profiles, 32 of which were full profiles and 2 partial profiles. MiniFiler PCR Amplification Kit produced 40 out of 49 DNA profiles and only 2 were partial. Identifiler PCR Amplification Kit was used for 4 skeletons, and 2 full and 2 partial profiles were obtained. DNA was also extracted from 23 blood samples of the living relatives.

**Table 3:** Summary of DNA analysis data for the skeletal remains from the island of Daksa

Characteristics	
Exhumed remains	53
Obtained DNA profiles	49
Ratio of obtained profiles (%)	92.43
Relatives' samples	23
Positive identifications	18
Ratio of positive identifications (%)	33.96
Ratio of positive identifications (%) regarding the number of requesting families	78.26

Cross-matching of the results with the relatives’ data resulted in 18 positive identifications. In 4 cases, the victims were identified by using only the autosomal STR systems for identification because positively identified remains belonged to the fathers of the daughters who gave the blood sample. The results obtained by MiniFiler PCR Amplification Kit were confirmed by the Identifiler PCR Amplification Kit, which allows simultaneous amplification of 15 autosomal STR loci, whereas MiniFiler PCR Amplification Kit gives the profiling result for 8 autosomal loci (both kits include sex-determining locus amelogenin).

**Figure 10:**
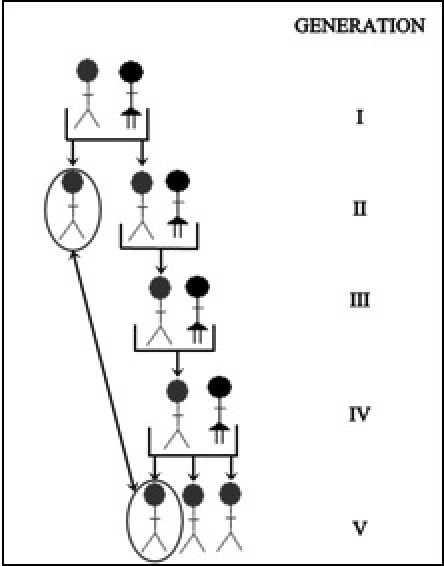
Schematic presentation of positive identification in a case where great-grandfather's brother was identified by matching with great-grandson's paternally inherited Y chromosome.

**Figure 11:**
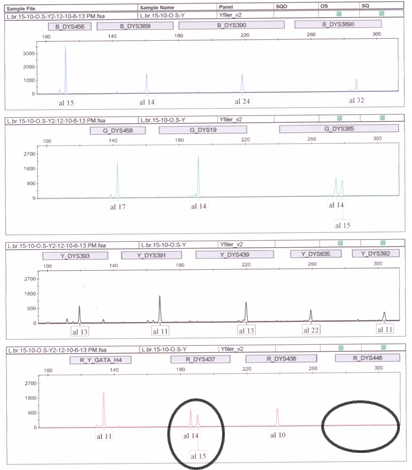
Y chromosome short tandem repeat loci mutations in a father/son pair with double peaks at DYS437 locus and with null allele at DYS448 locus (in circles).

In other 14 cases, the victims were identified by the Y-chromosomal STR system because the victims were paternally related to the individuals who gave blood samples. Nine positive identifications were made for father/son pairs, 3 for nephew/uncle pairs, 1 for grandchild/grandfather pair, and 1 for great-grandson/great-grandfather’s brother pair (Figure 10). We also found rare Y chromosome STR loci mutations in a father/son pair with double peaks at DYS437 locus, as well as with the null allele at DYS448 locus observed in the same father/son pair (Figure 11).

## Discussion

This study presents the anthropological, forensic, and genetic analysis of victims from the mass grave on the island of Daksa, near Dubrovnik. The remains were found to belong to at least 53 distinctive victims. All were male, mostly with gunshot wounds to the head. DNA analysis and cross-matching of the samples with relatives resulted in 14 positive identifications using the Y-chromosomal STRs and 4 positive identifications using the autosomal STRs. Forensic analysis revealed that most persons with identified gunshot wounds to the head died a violent death due to those wounds. The same applies to the persons with identified peri-mortem skull fractures and fragmented skulls. Arm bones and fore-arm bones tied up with wire indicate that this was a mass execution grave. The manner of the burial and particularly the localization of the penetrating gunshot wounds of 9 mm in diameter and the 9 mm pistol bullets with corresponding shells found at the site also suggest that this was an execution. The persons executed were probably in the kneeling position at the burial site or in its immediate vicinity. The objects found at the site indicate that they were mostly civilians. Also, according to the items found in the mass grave and to the results of DNA identification, at least 3 victims were priests.

One of the main problems in excavations of the mass graves dating from the World War II is a large number of damaged and separated skeletal remains (14). The excavations are usually complicated by environmental factors, such as difficult access to the site, subsequently raised constructions, vegetation, and often only assumed micro-location of the gravesite (15). Financing, manpower issues, the available time, and particularly the lack of experienced and educated teams also have a significant impact on the methodology of excavation.

The main objective of the World War II mass graves exhumation is to determine the minimum number of victims (if not always the precise), identify the cause and manner of death, and carry out the identification test, if possible, and return the remains to their families, in accordance with the Geneva conventions (16).

Since the bones of a large number of victims were found separated and intermingled, often in piles, a fast and bulk approach is satisfactory, particularly if the grid use (network) and the orientation of long bones at the site is carefully observed. Such method provides a significant saving of time and resources and still gives necessary answers to the questions raised. With the methods described above and additional work in the pathology room and at the desk, it is possible to subsequently reconstruct positions and relations of the remains by combining the data collected during the exhumation with the basic anthropometrics data revealed in the pathology room.

Anthropometrics data and the teeth status have an extremely limited role in the identification of mass graves victims dating from the World War II, mostly because there are no ante-mortem data related to victims (17,18). There was a relatively limited number of citizens of Dubrovnik who were expected to be found on the island of Daksa, but after more than 60 years no relevant data on their physical characteristics (more accurate than short/tall) or the teeth status could have been obtained, although a large number of victims had significant prosthetics works. There were no significant problems in the anthropological determination of the victims' sex, whereas the age determination (especially for the older ones) was not sufficiently precise for identification. We were able to determine the actual age of the victims only after the DNA analysis and positive identification, according to the information given by the relatives (19).

It was easy to identify the cause and the manner of death, since the execution with a single shot to the back of the head was a common manner of execution in mass graves victims from the World War II discovered in this region, as was the burial at the place of the execution (17).

A well-organized field work and careful proceedings in the pathologist room can “tell the story” about the last moments of the victims’ lives, while the identification of victims is exclusively in the domain of the DNA analysis (14,17,20). The latter was the imperative in our research, and was mostly done through Y chromosome analysis of male skeletal remains.

We successfully identified 14 persons by the AmpFiSTR Yfiler PCR Amplification Kit. One of them was an honorable Croatian priest, reverend Petar Perica, known as the author of some of the most beautiful Croatian religious songs (Box 1). His identification was confirmed by the Y-chromosome STR profile compatibility with the blood sample of his brother’s great-grandson.

Box 1 **Croatian religious poem written by Rev. Petar Perica, one of the identified victims**

Heavenly Virgin, Queen of the Croats

(*Rajska djevo kraljice Hrvata*)

Hail, Mary, full of all graces, Eternal sunshine, clad in radiance, Circling your brow, a starry crown, Below your feet, hell’s dragon groans. Heavenly Virgin, Queen of the Croats, Our Mother, Our Golden Dawn, From devoted hearts, receive a gift, Receive our pure and fervent love. Blessed are you, all Immaculate, The serpent’s breath taints not your breast! Star of happiness, resplendent for us, Disperse evil darkness, nights of sin!

http://www.youtube.com/watch?v=I_SC8_SGHv0

While observing a null Y-STR locus should not represent a problem for the profile interpretation, more than one peak at one or more loci could lead to a grave misinterpretation of a profile and its sample source. The same rare mutations and their interpretation have been already reported (21). Multiple variations (deletion or duplications), even if rare, can happen in the same single-source sample and these examples should be a reminder before drawing premature conclusions. Forensic interpretation of Y-haplotype profiles should be in the focus because multiple alleles at various loci do not necessarily indicate that the sample originates from a mixture. This also showed the importance of the DNA identification methods and their application in the case work.

This study showed that even in cases of more than 50-year-old, highly degraded human remains from mass graves, Y-chromosomal and autosomal STRs analysis can contribute to the identification of the victims.

## References

[1] Radica J. All our Daksas [in Croatian]. Dubrovnik (Croatia): Matica hrvatska; 2003.

[2] Primorac D. Application of DNA analysis in forensic medicine and judicature [in Croatian]. Zagreb: Pravo, Nakladni zavod Matice hrvatske; 2001.

[3] Primorac D, Andelinovic S, Definis-Gojanovic M, Drmic I, Rezic B, Baden MM (1996). Identification of war victims from mass graves in Croatia, Bosnia and Herzegovina by use of standard forensic methods and DNA typing.. J Forensic Sci.

[4] Andelinovic S, Sutlovic D, Erceg Ivkosic I, Skaro V, Ivkosic A, Paic F (2005). Twelve-years experience in identification of skeletal remains from mass graves.. Croat Med J.

[5] Trotter M, Gleser GC (1977). Corrigenda to «estimation of stature from long limb bones of American Whites and Negroes».. Am J Phys Anthropol.

[6] Slaus M, Strinovic D, Skavic J, Petrovecki V (2003). Discriminant function sexing of fragmentary and complete femora: standards for contemporary Croatia.. J Forensic Sci.

[7] Brooks S, Suchey JM (1990). Skeletal age determination based on the os pubis. A comparison of the Assadi-Nemeskeri and Suchey-Brooks methods.. Hum Evol.

[8] Alonso A, Andelinovic S, Martin P, Sutlovic D, Erceg I, Huffine E (2001). DNA typing from skeletal remains: evalution of multiplex and megaplex STR systems on DNA isolated from bone and teeth samples.. Croat Med J.

[9] Walsh PS, Metzger DA, Higuchi R (1991). Chelex 100 as a medium for simple extraction of DNA for PCR-based typing from forensic material.. Biotechniques.

[10] Applied Biosystems. AmpFlSTR Y Filer PCR Amplification Kit User Manual. Foster City (CA): Applied Biosystems; 2006.

[11] Mulero JJ, Chang CW, Lagace RE, Wang DY, Bas JL, McMahon TP (2008). Development and validation of the AmpFlSTR MiniFiler PCR Amplification Kit: a MiniSTR multiplex for the analysis of degraded and/or PCR inhibited DNA.. J Forensic Sci.

[12] Primorac D, Schanfield MS (2000). Application of forensic DNA testing in the legal system.. Croat Med J.

[13] Butler JM. Forensic DNA typing: Biology, technology and genetics of STR markers, 2nd edition. London: Elsevier Academic Press; 2005.

[14] Palo JU, Hedman M, Soderholm N, Sajantila A (2007). Repatriation and identification of Finnish World War II soldiers.. Croat Med J.

[15] Jessee E, Skinner M (2005). A typology of mass grave and mass grave-related sites.. Forensic Sci Int.

[16] United Nations Office of the High Commissioner for Human Rights. Protocol additional to the Geneva Conventions of 12 August 1949, and relating to the Protection of Victims or International Armed Conflicts (Protocol 1). Available from: http://www.icrc.org/ihl.nsf/7c4d08d9b287a42141256739003e636b/f6c8b9fee14a77fdc125641e0052b079 Accessed: June 3, 2011.

[17] Definis-Gojanović M, Sutlović D (2007). Skeletal remains from World War II mass grave: from discovery to identification.. Croat Med J.

[18] Hollmann T, Byard RW, Tsokos M (2008). The processing of skeletonized human remains found in Berlin Germany.. J Forensic Leg Med.

[19] Djuric M, Dunjic D, Djonic D, Skinner M (2007). Identification of victims from two mass graves in Serbia: a critical evaluation of classical markers of identity.. Forensic Sci Int.

[20] Zupanic Pajnic I, Gornjak Pogorelc B, Balazic J (2010). Molecular genetic identification of skeletal remains from the Second World War Konfin I mass grave in Slovenia.. Int J Legal Med.

[21] Decker AE, Kline MC, Redman JW, Reid TM, Butler JM (2008). Analysis of mutations in father-son pairs with 17 Y-STR loci.. Forensic Sci Int Genet.

